# Genome-scale metabolic reconstruction and *in silico *analysis of methylotrophic yeast *Pichia pastoris *for strain improvement

**DOI:** 10.1186/1475-2859-9-50

**Published:** 2010-07-01

**Authors:** Bevan KS Chung, Suresh Selvarasu, Camattari Andrea, Jimyoung Ryu, Hyeokweon Lee, Jungoh Ahn, Hongweon Lee, Dong-Yup Lee

**Affiliations:** 1 NUS Graduate School for Integrative Sciences and Engineering, National University of Singapore, 28 Medical Drive, #05-01, 117456, Singapore; 2 Bioprocessing Technology Institute, Agency for Science, Technology and Research (A*STAR), 20 Biopolis Way, #06-01, Centros, 138668, Singapore; 3 Biotechnology Process Engineering Center, KRIBB, Daejeon 305-806, Republic of Korea; 4 Department of Chemical and Biomolecular Engineering, National University of Singapore, 4 Engineering Drive 4, 117576, Singapore

## Abstract

**Background:**

*Pichia pastoris *has been recognized as an effective host for recombinant protein production. A number of studies have been reported for improving this expression system. However, its physiology and cellular metabolism still remained largely uncharacterized. Thus, it is highly desirable to establish a systems biotechnological framework, in which a comprehensive *in silico *model of *P. pastoris *can be employed together with high throughput experimental data analysis, for better understanding of the methylotrophic yeast's metabolism.

**Results:**

A fully compartmentalized metabolic model of *P. pastoris *(*iPP*668), composed of 1,361 reactions and 1,177 metabolites, was reconstructed based on its genome annotation and biochemical information. The constraints-based flux analysis was then used to predict achievable growth rate which is consistent with the cellular phenotype of *P. pastoris *observed during chemostat experiments. Subsequent *in silico *analysis further explored the effect of various carbon sources on cell growth, revealing sorbitol as a promising candidate for culturing recombinant *P. pastoris *strains producing heterologous proteins. Interestingly, methanol consumption yields a high regeneration rate of reducing equivalents which is substantial for the synthesis of valuable pharmaceutical precursors. Hence, as a case study, we examined the applicability of *P. pastoris *system to whole-cell biotransformation and also identified relevant metabolic engineering targets that have been experimentally verified.

**Conclusion:**

The genome-scale metabolic model characterizes the cellular physiology of *P. pastoris*, thus allowing us to gain valuable insights into the metabolism of methylotrophic yeast and devise possible strategies for strain improvement through *in silico *simulations. This computational approach, combined with synthetic biology techniques, potentially forms a basis for rational analysis and design of *P. pastoris *metabolic network to enhance humanized glycoprotein production.

## Background

In the biopharmaceutical industry, over 70% of the therapeutic proteins under preclinical and clinical development are glycosylated and there has been an increasing need for highly efficient glycoprotein expression systems. Mammalian systems such as Chinese hamster ovary (CHO) cells have been most widely used since they have been extensively characterized and are capable of human-like glycosylation. However, they typically exhibit low survivability and low recombinant protein productivity unless sophisticated experimental techniques were employed [1]. On the other hand, although yeast systems typically produce hyper-mannosylated proteins with poor bioactivity in humans, recent advances in yeast glycoengineering, especially for *Pichia pastoris*, enabled the synthesis of humanized glycoproteins with substantially improved bioactivity [2,3]. Furthermore, since the scale-up of production in yeast is a well-established technology, we can potentially achieve cost-effective and high-throughput production of therapeutic glycoproteins [2,3]. Thus, the methylotrophic yeast *P. pastoris *is expected to be one of the promising hosts for industrial production of recombinant protein in the near future. Indeed, companies are already beginning to use *P. pastoris *for the production of several therapeutic proteins [4,5].

A number of studies have been carried out for understanding and enhancing physiological properties of *P. pastoris*. These studies have elucidated various favorable characteristics of the organism, which makes it more attractive for large-scale production of recombinant humanized glycoprotein [6-10]. In particular, the lower tendency of hyper-mannosylation of proteins and negative Crabtree phenotype can present *P. pastoris *as a more superior expression host than the well-characterized *Saccharomyces cerevisiae *[7,11]. Hence, it is highly desirable to make significant efforts for improving the *P. pastoris *strain in order to achieve economic feasibility and efficiency [12-14]. In this regard, the emerging paradigm of systems biotechnology can play an important role in identifying key targets for strain improvement [15].

Systems biotechnology integrates high-throughput omics data and *in silico *modeling and analysis to understand and design cellular system to achieve desirable properties [16]. This approach has been successfully applied to one of the best-characterized expression system, *S. cerevisiae*, which was metabolically engineered for improved production of chemicals and biopharmaceuticals [17,18]. For example, targets for genetic manipulation have been identified to enhance the production of bioethanol [19,20] and human superoxide dismutase [21]. Therefore, we can yield similar benefits for *P. pastoris *by resorting to the systems biotechnological approach, which requires a comprehensive *in silico *metabolic model [22].

To date, more than 50 genome-scale metabolic models have been reconstructed for over 30 species from three main domains of life, i.e. Archaea, Bacteria and Eukarya [23,24]. Various applications of genome-scale metabolic models have been reported for characterizing cellular metabolism and guiding metabolic engineering. Examples include characterization of various organisms ranging from unicellular bacteria such as *Escherichia coli *[25] and *Zymomonas mobilis *(Widiastuti H, Kim JY, Selvarasu S, Karimi IA, Kim H, Seo JS, Lee DY: Genome-scale modeling and *in silico *analysis of ethanologenic bacteria *Zymomonas mobilis*, submitted) to complex mammalian systems such as *Mus musculus *[26] and even *Homo sapiens *[27]; phenotypic prediction of metabolic-gene deletion strains of *E. coli *[28]; and identification of metabolic engineering targets to enhance biochemical production capability of *E. coli *[29,30]. Towards this end, we reconstructed a genome-scale model of *P. pastoris *metabolism which allows us to elucidate interesting features of the methylotrophic yeast and identify engineering targets for achieving enhanced physiological properties of the strain.

## Results

### Characteristics of reconstructed metabolic network

**Figure 1:**
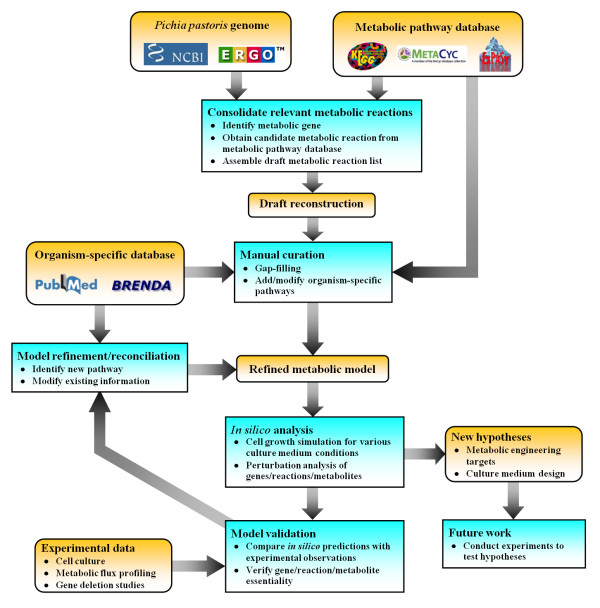
**Reconstruction of *Pichia pastoris***. A recently published genome of *P. pastoris *[35] and various online databases, including KEGG [71], MetaCyc [70], PubMed http://www.ncbi.nlm.nih.gov/pubmed/, BRENDA [68] and ExPASy ENZYME database [69], were used to reconstruct and manually curate the *iPP*668 metabolic model.

Following the procedure illustrated in Figure 1, a genome-scale network of *P. pastoris *metabolism was reconstructed (see Methods). During the reconstruction process, significant efforts were required in the gap-filling process. Essential metabolic reactions are added sequentially to the draft model, through repeated cell growth simulations using constraints-based flux analysis, to ensure *in silico *viability. For example, a metabolic gap exists in the initial draft of the methonine biosynthetic pathway as the gene coding for the enzyme catalyzing transamination of 2-keto-4-methylbutyrate to form methionine [31] was not found and had to be added manually. Similarly, in order to allow mitochondrial localization of acyl-carrier protein for fatty acid synthesis, a mitochondrial transport reaction was introduced to the model, thus improving metabolic network connectivity.

The reconstructed metabolic model of *P. pastoris*, *iPP*668, accounts for 668 metabolic genes, 1,361 reactions and 1,177 metabolites segregated into 8 compartments: cytosol, endoplasmic reticulum, extracellular matrix, Golgi apparatus, mitochondria, nucleus, peroxisome and vacuole as summarized in Table 1 (see Additional file 1 for model details). The metabolic reactions include 904 intra-compartment reactions, 308 inter-compartment reactions, and 149 extracellular exchange reactions. These metabolic reactions are also classified into 62 subsystems (Table 2) which include pathways in central carbon metabolism, amino acid, carbohydrate and lipid biosynthetic pathways, and more notably, the methanol utilization pathway previously characterized by [32]. In the fully compartmentalized model, metabolites localized in different cellular compartments (e.g. cytosolic pyruvate, pyr[c] and mitochondrial pyruvate, pyr[m]) are considered as distinct metabolites. Thus, without considering sub-cellular compartmentalization, this model accounts for 681 unique metabolites.

**Table 1:** Summary statistics of metabolic reconstruction for two yeast models

Genome characteristics	*P. pastoris*		*S. cerevisiae*	
Genome length	9.3 Mbp		12.1 Mbp	
G+C content	41.1%		38.3%	
Coding genes	5,313		6,607	
				
Model characteristics				
No. of genes	668		904	
No. of gene-associated reactions	1,007		1,043	
No. of other reactions	354		534	
No. of internal reactions	1,212		1,413	
No. of exchange reactions	149		164	
No. of metabolites	1,177		1,228	
				
**Compartment**	**No. of metabolites**	**No. of reactions**	**No. of metabolites**	**No. of reactions**
Cytosol	607	623	634	709
Endoplasmic reticulum	28	15	28	15
Extracellular matrix	149	12	164	14
Golgi apparatus	15	4	17	6
Mitochondria	235	163	241	175
Nucleus	35	16	40	16
Peroxisome	84	66	80	65
Vacuole	24	3	24	3
Inter-compartment	-	452	-	574

**Total**	**1,177**	**1,361**	**1,228**	**1,577**

Data of *S. cerevisiae *is obtained from [37] and *Saccharomyces *Genome Database http://www.yeastgenome.org/.

**Table 2:** Functional classification of metabolic reactions

Alanine and Aspartate Metabolism	9	Other Amino Acid Metabolism	10
Alternate Carbon Metabolism	21	Oxidative Phosphorylation	18
Anaplerotic reactions	10	Pantothenate and CoA Biosynthesis	16
Arabinose Metabolism	3	Pentose Phosphate Pathway	13
Arginine and Proline Metabolism	31	Phospholipid Biosynthesis	42
Asparagine metabolism	2	Phospholipid Metabolism	8
ATP maintenance	1	Porphyrin and Chlorophyll Metabolism	13
Biomass requirement	1	Purine and Pyrimidine Biosynthesis	51
Citric Acid Cycle	13	Pyridoxine Metabolism	8
Complex Alcohol Metabolism	22	Pyruvate Metabolism	17
Cysteine Metabolism	7	Quinone Biosynthesis	17
Fatty Acid Biosynthesis	61	Riboflavin Metabolism	11
Fatty Acid Degradation	42	Sphingolipid Metabolism	57
Fatty Acid Metabolism	3	Starch and Sucrose Metabolism	2
Folate Metabolism	23	Sterol Metabolism	48
Fructose and Mannose Metabolism	8	Taurine Metabolism	1
Galactose metabolism	2	Thiamine Metabolism	12
Glutamate metabolism	15	Threonine and Lysine Metabolism	16
Glutamine Metabolism	3	Transport, Endoplasmic Reticular	8
Glycerolipid Metabolism	11	Transport, Extracellular	145
Glycine and Serine Metabolism	19	Transport, Golgi Apparatus	2
Glycolysis/Gluconeogenesis	22	Transport, Mitochondrial	91
Glycoprotein Metabolism	5	Transport, Nuclear	5
Histidine Metabolism	14	Transport, Peroxisomal	20
Methanol Metabolism	7	Transport, Vacuolar	25
Methionine Metabolism	18	tRNA charging	35
NAD Biosynthesis	18	Tyrosine, Tryptophan, and Phenylalanine Metabolism	34
Nitrogen Metabolism	3	Valine, Leucine, and Isoleucine Metabolism	19
Nucleotide Salvage Pathway	61	Xylose Metabolism	2
Other	11	Exchange reactions	149
		**Total**	**1361**

The table shows the number of reactions classified under each subsystem.

In *iPP*668, we have included a biomass equation which is composed of biosynthetic precursors and energy requirement. Appropriate coefficients of amino acids and carbohydrates were obtained from a recent experimental composition analysis of *P. pastoris *[33] while lipid and sterol compositions were evaluated based on an analytical study of the cellular membranes [34]. The reported G+C content of 41.1% [35] forms the basis of our calculation of nucleotide composition and the composition of the individual RNA is assumed to be similar to that of *S. cerevisiae *[36] according to findings by [33]. We also calculated the growth associated ATP requirement for the polymerization of proteins, DNA and RNA and included this energy requirement as a part of the biomass synthesis equation (see Additional file 2 for details).

**Figure 2:**
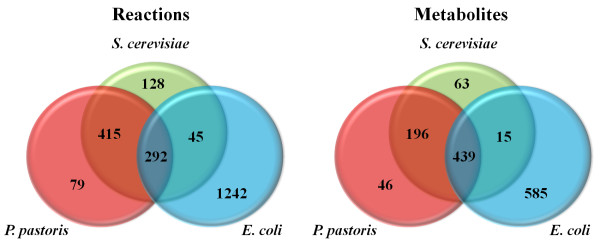
**Comparison of metabolic model reconstructions**. The *P. pastoris *metabolic model reconstruction *iPP*668 is being compared with *S. cerevisiae *[37] and *E. coli *[25] and it is found that there is no reaction that is shared by *E. coli *and *P. pastoris*. The number in each section of the Venn diagram indicates the number of reactions that are common or specific to the respective organism(s).

**Figure 3:**
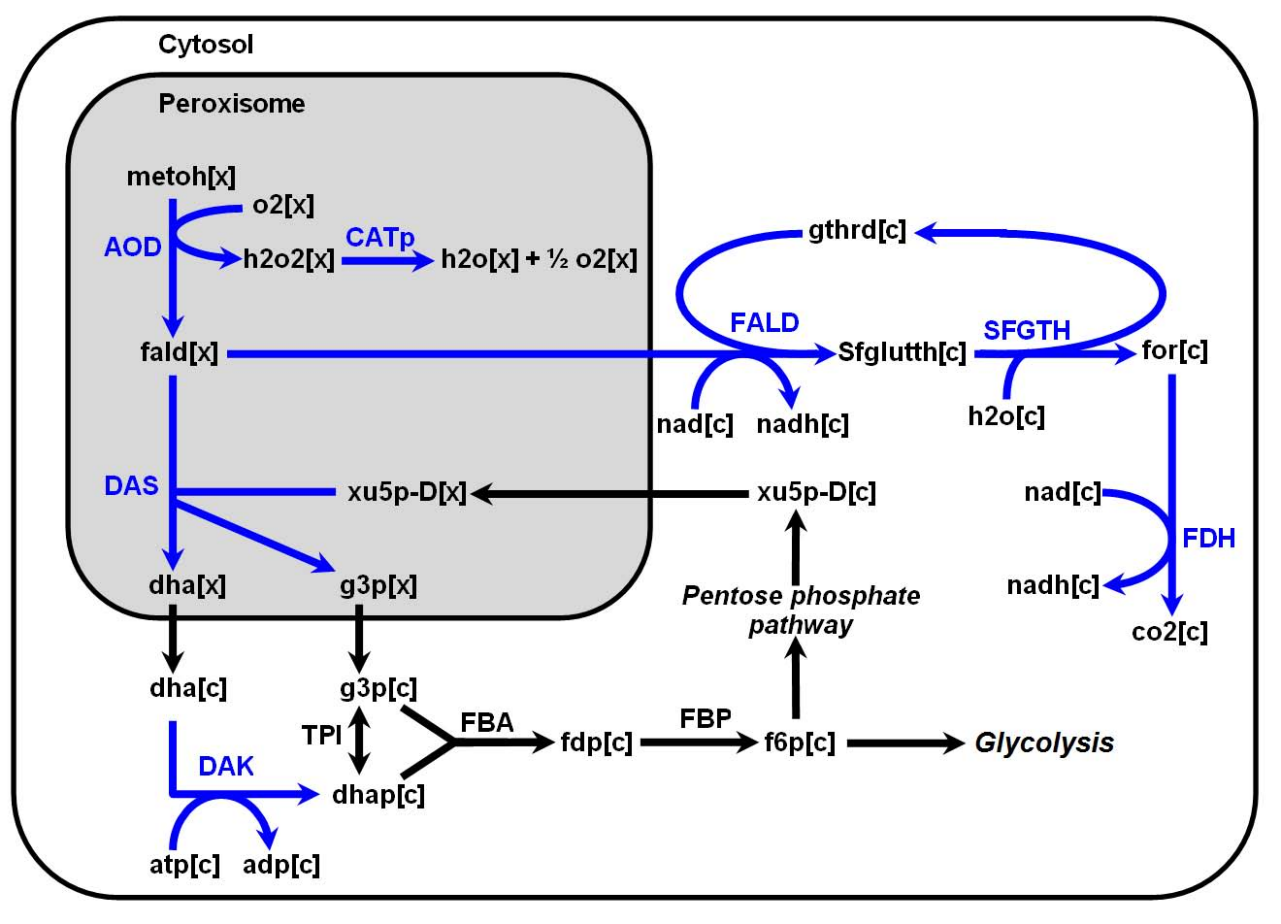
**Methanol utilization pathway**. Reactions unique to *P. pastoris *are highlighted in blue. Metabolites such as water, protons, phosphate are omitted (see Additional file 1 for abbreviation and reaction details).

Unique and conserved features in *P. pastoris *metabolism were further elucidated by comparing *iPP*668 with two model organisms: *S. cerevisiae *(*i*MM904) [37] and *E. coli *(*i*AF1260) [25]. It should be noticed that we disregarded the sub-cellular compartmentalization of reactions for the comparative analysis of metabolic capability by eliminating inter-compartmental transport and metabolic reaction duplicates in different compartments. From this comparison, there are 292 reactions and 439 metabolites that are common to the three species (Figure 2). These reactions largely belong to the central carbon metabolism and amino acid biosynthetic pathways. The 415 reactions and 196 metabolites, shared only by *P. pastoris *and *S. cerevisiae*, are generally classified under the lipid and carbohydrate biosynthetic pathways while the 79 reactions and 46 metabolites unique to *P. pastoris *are mainly from the methanol utilization pathway (Figure 3) and certain parts of lipid metabolism which are yeast-specific. It is further observed that the lipid biosynthetic pathways of both yeasts are structurally identical, only differing in the composition of fatty acid chains.

### Validation of phenotypic predictions

In this study, we validated the *iPP*668 metabolic model by checking the difference between predicted cellular phenotypes and experimental observations obtained from two sets of chemostat culture conditions, glucose minimal medium (see Methods) and glycerol/methanol mixed medium [38]. In both cases, we maximized cell growth while constraining carbon source uptake rates at experimentally determined levels.

**Figure 4:**
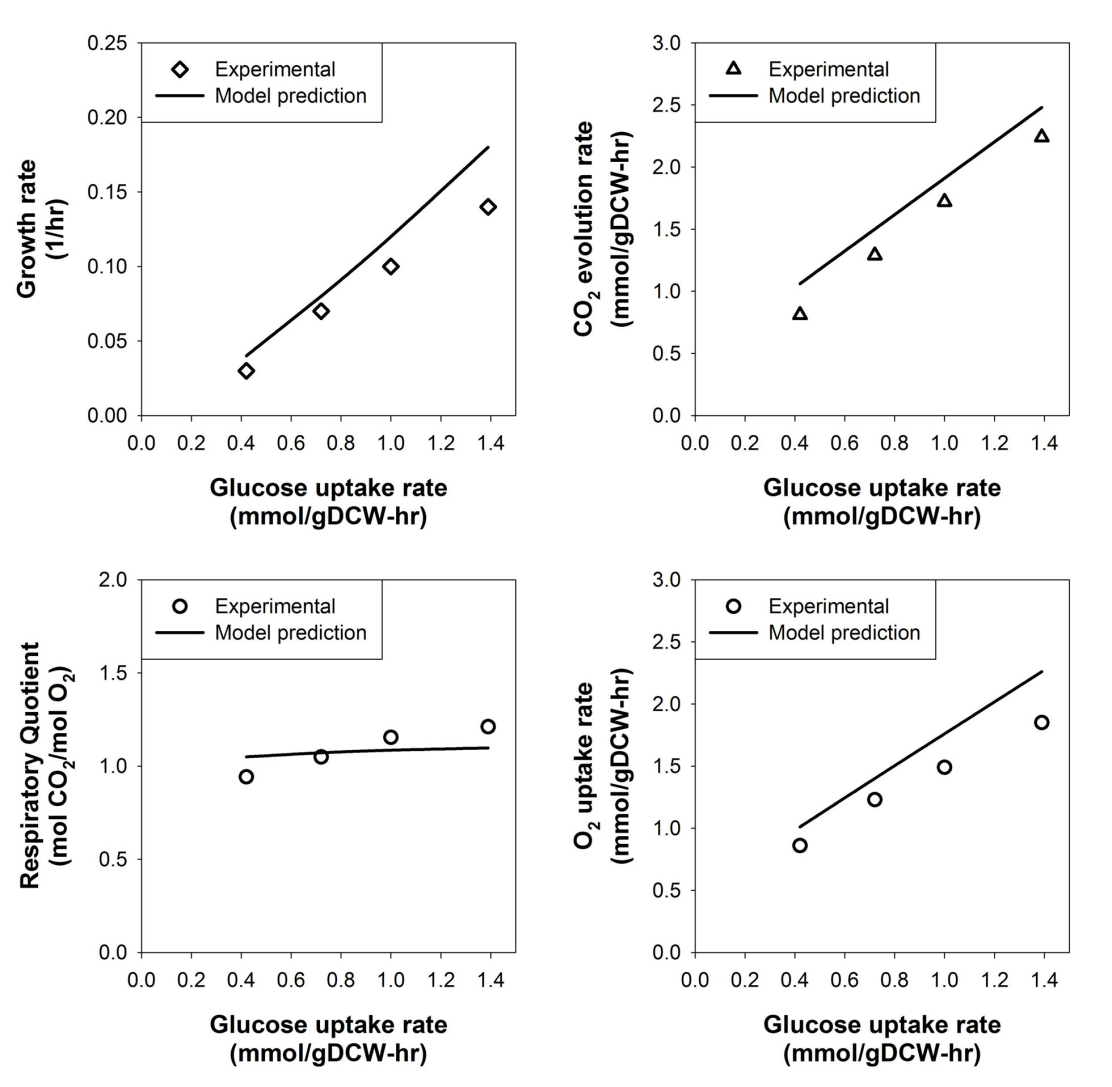
**Chemostat culture simulation results**. Data points for each chemostat experimental data point were generated by constraints-based flux analysis implemented with glucose uptake rate constrained at the respective experimentally determined values.

In the case of glucose minimal medium, we observed that the predicted cell growth, oxygen uptake and carbon dioxide evolution rates are highly consistent with the data obtained from our chemostat experiments (Figure 4). Discrepancies between experimental observations and model predictions are within 20% which can be considered acceptable. The constantly higher growth and gaseous exchange rate predictions can be due to possible errors in biomass composition data obtained from various sources (see Methods and Additional file 2). It should be noted that in order to mimic physiological states, we specified conditional parameters (see Methods). NGAM requirement (2.3 mmol ATP/gDCW-hr) was estimated from our chemostat data whereas we defined P/O ratio (1.48 mol ATP/mol O) based on previous studies [39,40].

Another set of chemostat culture data [38] also allowed us to evaluate the fidelity of physiological predictions under the glycerol/methanol mixed media condition. In this case, the NGAM requirement is estimated to be about 6 mmol ATP/gDCW-hr while the P/O ratio remains unchanged. Predictions for cell growth in glycerol minimal media are reasonably consistent with experimental observations but the discrepancy becomes significant when methanol uptake rate is high (Table 3). In the latter case, methanol uptake induces the synthesis of alcohol oxidase enzyme and generation of peroxisome lipid membrane which may cause substantial alteration in the cellular composition of amino acids and lipids [7,41]. Moreover, the effects of metabolic regulation, such as the repression of *AOX *by glycerol [42], that are not captured in the current model may also contribute to the discrepancy. Thus, future works in the experimental analysis of changes in biomass composition during methanol induction can provide necessary information to improve prediction capability of the current metabolic model.

**Table 3:** *In silico *prediction of growth in glycerol/methanol mixtures

	Dilution rate, *D*							
	*D *= 0.16		*D *= 0.16		*D *= 0.05		*D *= 0.05	
	**Exp **^a^	Pred	**Exp **^a^	Pred	**Exp **^a^	Pred	**Exp **^a^	Pred
**Cell growth**	0.16	0.18	0.16	0.19	0.05	0.05	0.05	0.05
**Glycerol uptake **^b^	2.75		2.23		1.09		0.57	
**Methanol uptake **^b^	-		2.73		-		2.33	
**CO**_**2 **_**evolution**	2.35	2.40	3.60	3.22	1.56	1.72	2.21	2.37
**O**_**2 **_**uptake**	3.62	3.56	7.20	5.47	2.16	2.20	4.85	3.76
**Respiratory quotient**	0.65	0.68	0.50	0.59	0.72	0.78	0.46	0.63
**P/O ratio**	-	1.48	-	1.48	-	1.47	-	1.49

In the chemostat culture, growth rate is equivalent to dilution rate. Units of measurements are given as follows: growth/dilution rate, 1/hr; carbon source uptake or gaseous exchange rates, mmol/gDCW-hr; Respiratory quotient, mol CO_2_/mol O_2_; P/O ratio, mol ATP/mol O.

^a ^Data from [38].

^b ^Carbon source uptake rates are model input parameters which are not predicted.

### Carbon sources for recombinant protein production

Among the various carbon sources available, methanol is the inducer for *AOX *promoter which is commonly used for heterologous protein production in *P. pastoris*. However, the yeast typically grows very slowly on this carbon source [7]. Thus, an alternative substrate can be supplied during the initial phase to achieve high cell density, followed by shifting into methanol induction phase for protein production [43]. Based on this feeding strategy, desirable carbon sources must be able to yield high growth rate with high capacity to synthesize amino acids in the earlier phase so that available amino acid pools can be diverted to produce recombinant protein in the later phase. In this sense, we can explore the effect of various carbon sources (alanine, glucose, glycerol, methanol, sorbitol and trehalose) on cellular metabolism of *P. pastoris *using the reconstructed model and as such, identify the best candidate for recombinant protein production. To do so, we simulated cell growth by constraining the supply of each carbon source to one C-mmol/gDCW-hr (i.e. 0.167 mmol/gDCW-hr for glucose, 0.333 mmol/gDCW-hr for glycerol, 1 mmol/gDCW-hr for methanol, etc.).

**Figure 5:**
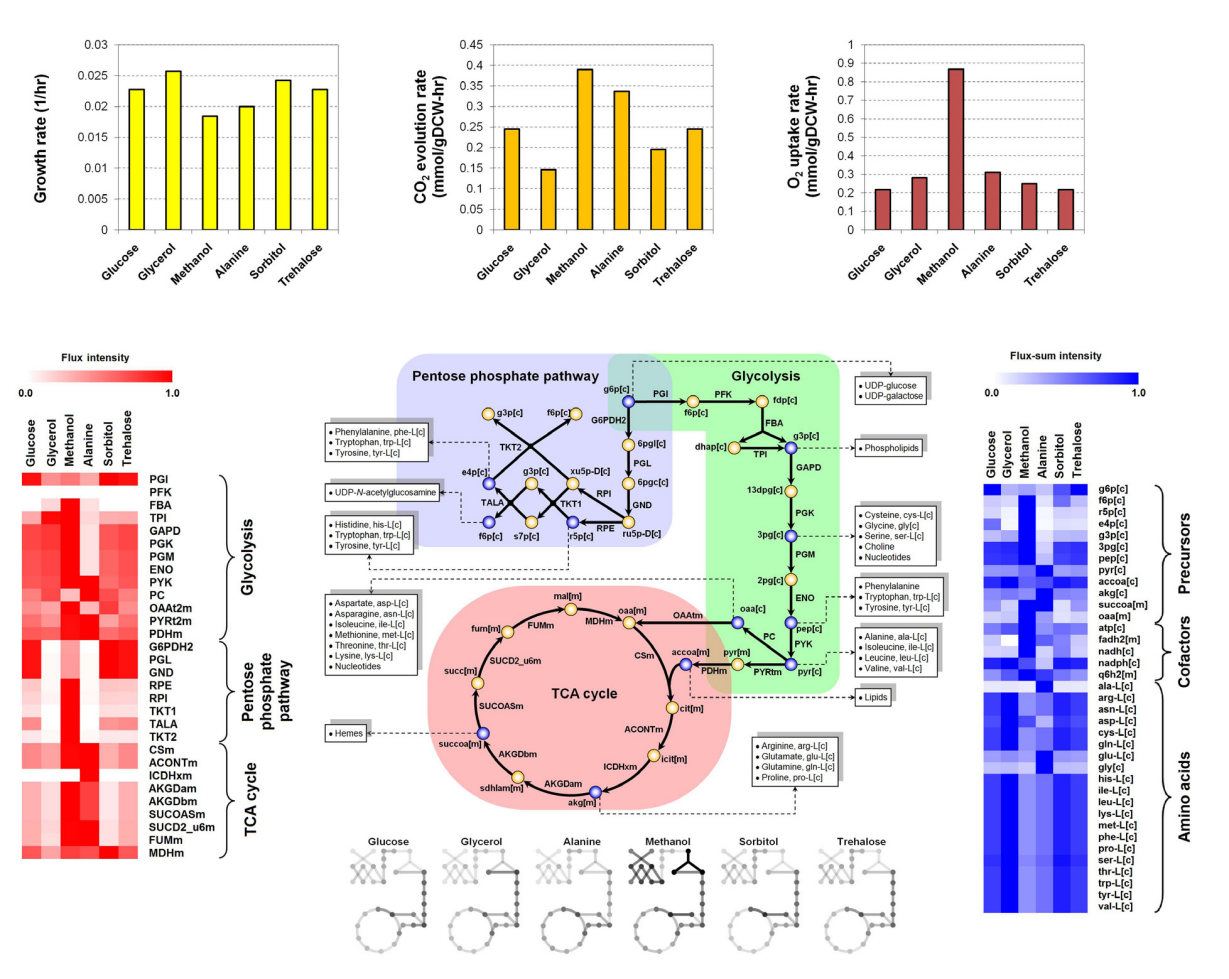
**Flux and flux-sum distributions for different carbon sources**. Growth rate, flux and flux-sum values were generated based on individual carbon source uptake rate of 1 C-mmol/gDCW-hr. The color intensity of the lines in the central carbon metabolic network corresponds to the flux values. Precursor metabolites are in blue and the building blocks derived from each of them are specified in the text boxes. The heat-maps on the left and right illustrate the flux and flux-sum distributions, respectively. Similarly, the color intensity corresponds to the flux or flux-sum values normalized with respect to the maximum for each reaction or metabolite.

Based on the first criterion of high growth yield, glycerol is the most promising candidate for recombinant protein production, followed by sorbitol (Figure 5). Generally, high utilization of the central metabolism can lead to increased production of various precursors, which can further synthesize building blocks required for the biomass. However, the resulting flux distributions indicated that the utilization of central carbon metabolism is the highest for methanol uptake despite yielding the lowest growth rate. We can understand this observation by examining gaseous exchange rates and ATP flux-sum for methanol utilization. The significantly higher turnover rate of ATP and gaseous exchange rates suggest that much of the resources have been diverted to energy generation during methanol utilization. The low respiratory quotient resulting from this diversion of resources is consistent with findings from several experimental studies [38,44]. Hence, the higher utilization of central metabolism is a consequence of the high energy requirement of methanol metabolism in *P. pastors*.

Not surprisingly, flux-sum distribution profiles on various carbon sources confirmed that sorbitol and glycerol are capable of generating higher amino acid flux-sum compared to others. Thus, this large pool of amino acids can potentially be diverted to synthesize recombinant protein during the induction phase, indicating sorbitol and glycerol are desirable carbon sources for recombinant protein production in terms of both growth yield and amino acid biosynthetic capability (Figure 5). However, if *AOX *promoter is used for recombinant protein expression, sorbitol will be more superior as glycerol is known to repress the promoter [42]. Therefore, the current model-driven evaluation of various carbon sources recommends a feeding strategy of supplying sorbitol as the co-substrate during methanol induction phase to provide the necessary resources for recombinant protein production.

### Application of *P. pastoris *for whole-cell biotransformation

Apart from recombinant protein production through methanol induction, we managed to identify another potential application of culturing *P. pastoris *in methanol. From the *in silico *analysis, it was observed that growing *P. pastoris *in methanol minimal media can exhibit high turnover of NADH, implying the potential capability of *P. pastoris *for whole-cell biotransformation. For example, value-added chemical precursors, e.g. 2,3-butanediol, can be synthesized via enzymatic reduction of ketones, e.g. acetoin, which requires NADH or NADPH regeneration [45-49]. In this aspect, the high rate of NADH regeneration in *P. pastoris *is suitable for biocatalysis of butanediol dehydrogenase reaction to convert acetoin into 2,3-butanediol [50]. To achieve cost-effectiveness, we can further enhance the NADH regeneration of *P. pastoris *by identifying gene targets to be overexpressed through the modified flux analysis implementation (see Methods).

**Figure 6:**
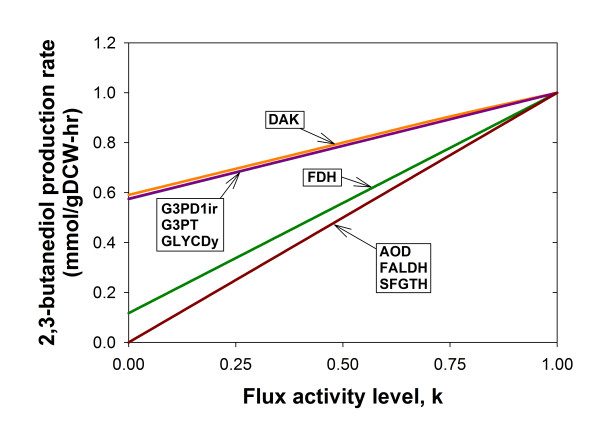
**Effect of flux activity on 2,3-butanediol production**. The flux activity level shown on the *x*-axis refers to the flux activity normalized with respect to the maximum flux activity value for each reaction. This value is also equivalent to the factor *k *used to carry out the analysis (see Methods). Reaction abbreviations: AOD, alcohol oxidase; DAK, dihydroxyacetone kinase; FALDH, formaldehyde dehydrogenase; FDH, formate dehydrogenase; G3PD1ir, glycerol-3-phosphate dehydrogenase; G3PT, glycerol-3-phosphatase; GLYCDy, glycerol dehydrogenase; SFGTH, S-formylglutathione hydralase (see Additional file 1 for details).

Simulation results show that when *P. pastoris *is supplied with 1 mmol/gDCW-hr of acetoin, attenuating the flux activity of some reactions can give rise to a proportional decrease in the maximum achievable butanediol dehydrogenase reaction flux (Figure 6). Fixing the fluxes of these reactions imposes an upper limit on the flux of butanediol dehydrogenase. Thus, such reactions are "directionally coupled" to 2,3-butanediol production, according to [51]. For the *in vivo *system, the directionally coupled reactions will become bottlenecks only if the corresponding enzymes were not expressed adequately while the maximum rate of 2,3-butanediol production may be achievable upon the expression of the enzymes at high levels. From this inference, the list of target enzymes identified can be genetically enhanced for relieving the bottleneck if the experimentally observed 2,3-butanediol production is significantly less than the theoretical value.

## Discussion

The genome-scale metabolic modeling and analysis procedure presented in this study can potentially be used to design culture media for enhancing the performance of *P. pastoris*. We have shown through the analysis of carbon source utilization that glycerol and sorbitol are the best candidates for achieving high growth yields in *P. pastoris *cell culture (Figure 5). Furthermore, with regards to recombinant protein production using the *AOX *promoter, sorbitol is clearly better than glycerol since the latter represses the *AOX *promoter [42]. The generation of high flux-sum for many of the amino acids due to sorbitol utilization (Figure 5) can provide a possible explanation for the successful use of sorbitol in recombinant protein expression experiments which utilizes methanol induction [52-55]. Although the technique of expressing recombinant protein using the *AOX *promoter has been successful, alternative promoters, such as gylceraldehyde 3-phosphate dehydrogenase (*GAP*), glutathione-dependent formaldehyde dehydrogenase (*FLD1*), peroxisomal matrix protein (*PEX8*) and secretion GTPase (*YPT1*) promoters, have been proposed since methanol, a petroleum compound, may not be perceived as an appropriate raw material for production of proteins for human consumption [56]. For the utilization of these alternative promoters, sorbitol may not be the best candidate due to regulatory factors and further analysis using *iPP*668 can provide us with the corresponding best carbon source selection.

Another application of *iPP*668 is the rational identification of metabolic engineering targets. From our analysis of carbon source utilization, we found that growing *P. pastoris *in methanol minimal medium is suitable for biocatalysis of ketone reduction such as the conversion of acetoin into 2,3-butanediol, which requires NADH regeneration [50]. In this case, the key enzyme, butanediol dehydrogenase, is overexpressed but the native metabolism of the host may consist of some limiting reactions or bottlenecks that prevent the maximum utilization of the butanediol dehydrogenase reaction. Some of the bottleneck reactions, AOD, FALD and FDH, identified by our *in silico *analysis have been demonstrated to be appropriate metabolic engineering targets for enhancing 2,3-butanediol production [50] and the other potential targets, DAK, G3PD1ir, G3PT and GLYCDy, can serve as hypotheses for future experimental validation. Hence, using the proposed computational framework, *iPP*668 can be a useful tool for tackling other metabolic engineering problems associated with *P. pastoris*.

Protein glycosylation is an important post-translational process which can affect the protein's secretion, folding and bioactivity [57,58]. Thus the extension of *iPP*668 to account for glycosylation processes can potentially increase the utility of the model. The design of glycosylation pathway through glycoengineering is critical for recombinant protein production in yeasts [3]. When combined with synthetic biology, effective glycoengineering techniques can even produce synthetic glycoproteins with enhanced therapeutic function [59]. Hence, it is highly desirable to develop a framework to rationally design the glycosylation pathway by analyzing the interaction between cellular metabolism and post-translational modification mechanisms, and subsequently optimizing the production of desired glycoforms, as shown in previous theoretical studies [60-63]. Although the limitations of constraints-based flux analysis prohibit detail modeling of the diverse range of glycoforms, it is still possible to account for the overall metabolic requirement involved in protein glycosylation as discussed by [26]. We can characterize the glycan structure of any target glycoprotein and evaluate the stoichiometry of monosaccharides required to synthesize it using advanced glycomics techniques [64]. We can then construct a lumped glycoprotein synthesis equation that also describes the energy requirement of glycosidic bond formation [65]. By adding this equation to the existing metabolic model, we can investigate the sensitivity of cellular metabolism to the carbohydrate and energy requirements of glycosylation. Consequently, this extended model offers a good platform for the integration of glycomics and other omics, such as metabolomics and fluxomics, for a large-scale systems analysis of the cellular physiology in *Pichia pastoris*.

During the peer review process of the current work, we identified another independent reconstruction effort that has been made by Sohn *et al*. [66], presenting the genome-scale model of *P. pastoris *metabolism, PpaMBEL1254. A model comparison between *iPP*668 and PpaMBEL1254 shows that both models are fully compartmentalized into 8 subcellular compartments, and capable of describing *P. pastoris *cellular metabolism under various carbon source uptake conditions. Nonetheless, several differences between the current study and Sohn *et al*. can be found with respect to the model size, validation and subsequent analysis. The size of *iPP*668 (668 genes, 1,361 reactions, 1,177 metabolites) is larger, providing more information than PpaMBEL1254 (540 genes, 1,254 reactions, 1,147 metabolites). In addition, *iPP*668 was validated by two sets of chemostat experimental data while Sohn *et al*. used batch fermentation data for qualitative model validation. Interestingly, Sohn *et al*. explored the capability of *P. pastoris *to produce recombinant proteins such as human serum albumin and human superoxide dismutase under various oxygenation rates using PpaMBEL1254, while we have discussed the general procedure to represent the protein production in the model based on amino acid biosynthetic rates. However, the PpaMBEL1254 model did not clearly describe the methanol utilization pathway of *P. pastoris *which is an important metabolic characteristic of great interest for applications in recombinant protein production and biotransformation, as discussed in the current study. Therefore, we suggest the combination of *iPP*668 and PpaMBEL1254 for future model expansion and systems metabolic engineering studies to harness the useful information provided by two genome-scale metabolic models.

## Conclusion

In this study, we have reconstructed a genome-scale metabolic model of the methylotrophic yeast *P. pastoris*, which has been developed for recombinant protein production as well as whole-cell biotransformation. The metabolic model was manually curated with information from literature and various databases to provide a good description of cell growth under various culture conditions through the use of constraints-based flux analysis. Validation of the curated model has shown that the model was able to reproduce key characteristics of *P. pastoris *metabolism reported in various experimental studies. The analysis of *P. pastoris *growth on different substrates has also allowed us to understand some useful application of the methylotrophic yeast and to generate testable hypotheses which can help to improve its biochemical production capability. Future works in the experimental analysis of *P. pastoris *can potentially enhance the predictive capability of the current model which can be integrated with high-throughput omics analysis for yeast systems biotechnology.

## Methods

### Reconstruction of metabolic model

The metabolic network of *P. pastoris *was reconstructed based on a recently published genome annotation of the organism [35]. An initial draft of the network is constructed by compiling annotated metabolic genes; relevant reactions are added with the respective gene-protein-reaction (GPR) assignments [23] based on information from online databases such as BioSilico [67], BRENDA [68], ExPASy ENZYME [69], MetaCyc [70] and KEGG [71] . The initial draft is subsequently refined through manual curation and gap-filling processes. In addition, constraints-based flux analysis was used to identify missing links that can be filled by adding necessary steps leading to the cell growth. The process of model refinement typically requires additional information from scientific references and publications which can be found in the PubMed database. Subsequently, relevant experimental validation and model analysis further refine the model in an iterative manner (Figure 1).

### Constraints-based flux analysis

Cellular metabolism can be simulated by constraints-based flux analysis as described elsewhere [72-74]. We used this approach to evaluate cell growth of *P. pastoris *under various culture conditions. Mathematically, the rate of biomass synthesis forms the objective function to be maximized, subjected to stoichiometric, thermodynamic and reaction capacity constraints, resulting in the following linear programming problem:

where *S*_*ij *_refers to the stoichiometric coefficient of metabolite *i *involved in reaction *j *and *v*_*j *_is the flux of reaction *j*. Reaction capacity and thermodynamic constraints, based on reaction reversibility information, are specified using the parameters *α*_*j *_and *β*_*j *_indicating the lower and upper bounds of reaction *j*, respectively. Unless experimental measurements are available, we typically specify *β*_*j *_= inf and *α*_*j *_= -inf (for reversible reactions) or *α*_*j *_= 0 (for irreversible reactions). In addition, gene deletion can be simulated by setting *α*_*j *_= *β*_*j *_= 0 for the associated metabolic reactions. In this study, the linear programming problem was solved using General Algebraic Modeling System (GAMS) Integrated Development Environment (IDE) version 22.7 [75].

### Stipulation of condition-specific parameters

The values of condition-specific parameters, such as non-growth associated ATP maintenance requirement (NGAM) and P/O ratio, vary with culture media and other environmental conditions. Thus, these values have to be determined for simulation of different experimental conditions. NGAM requirement is specified by fixing the flux value of ATP maintenance reaction. The experimental value is determined by plotting the uptake rates of carbon source against dilution rates from a chemostat run (see Additional file 2 for calculation details). P/O ratio is defined as the ratio of ATP produced to oxygen atom reduced by the respiratory chain. Thus, this value can be determined by calculating 0.5(*v*_ATPS3m_/*v*_CYOOm_) where ATPS3m and CYOOm refer to ATP synthase and cytochrome c oxidase reactions in mitochondria compartment, respectively. It can be changed by adjusting stoichiometric coefficients of hydrogen ions in the cytochrome c oxidase reaction. (See Additional file 1 for reaction details.)

### Describing metabolite turnover rate using flux-sum

The metabolic state of a cellular system can be described by the interconversion of metabolites. Here, the turnover rate of intermediate metabolites is defined as their flux-sum [76,77]. Since the overall consumption and generation rates are equal under the steady-state assumption, the flux-sum of metabolite *i *can be formulated as . Each |*S*_*ij*_*v*_*j*_| term in this summation series gives us the absolute rate of consumption/generation of metabolite *i *due to reaction *j *and thus by halving the sum of these terms, we can obtain the overall turnover rate for metabolite *i*.

### Identification of gene targets for overexpression

In metabolic engineering, bottlenecks in the metabolic network can be considered as potential gene targets to be overexpressed [78]. Therefore, it is important to devise a systematic framework for identifying such targets. In this regard, we modified the flux analysis procedure to detect relevant reactions that can potentially affect the biosynthetic rate of the product of interest (*v*_*t*arg*et*_). In our computational framework, *v*_*t*arg*et *_is maximized under different levels of reaction flux perturbation. Taking into consideration that the enhancement of biochemical production typically results in attenuated growth rate, we set the lower limit of cell growth to 50% of the maximum value (). The mathematical formulation can lead to the following mixed integer linear programming (MIP) problem:

We define "flux activity" as the absolute value of a reaction flux. Thus  and  refer to the maximum and minimum flux activity of reaction *j *respectively, under the cell growth constraint of . In the above formulation, big *M *is some arbitrarily chosen large value representatiing the flux upper limit which we typically specify as 1000 mmol/gDCW-hr. By solving the MIP problem for each reaction *j *and different values of *k *(in this case: 0, 0.25, 0.5, 0.75 and 1.0), we can evaluate the effect of individual reaction flux activity on the flux of any target reaction. The MIP is implemented and solved in GAMS IDE version 22.7 [75].

### Strain and chemostat culture

A wild type *Pichia pastoris *X-33 (Invitrogen, Carlsbad, CA, USA) was used for the chemostat culture. A single colony of the strain was inoculated into 20 mL of yeast protein dextrose (YPD) medium containing (per liter): 20 g of glucose, 10 g of yeast extract, and 20 g of Bacto-peptone and incubated overnight at 30°C. The culture was transferred to a 500 mL Erlenmeyer flask containing 100 mL of YPD broth and incubated overnight at 30°C. This culture was used as a seed for the chemostat culture using 2.5 L jar fermentors (KoBiotech, Incheon, Korea) with a constant volume of 800 mL. Cells were first batch-cultured in a defined glucose minimal medium containing (per liter): 50 g glucose, 1.5 g MgSO_4_·7H_2_O, 0.2 mg Biotin, 3 g KH_2_PO_4_, 15 g (NH_4_)_2_SO_4_, 2.0 mL of trace salts stock solution and 1.0 mL of a solution containing 2 g/L biotin in 1M NaOH. The trace element solution contained (per liter) 5 mL H_2_SO_4_, 4.0 g CuSO_4_·5H_2_O, 14.0 g MnSO_4_·H_2_O, 2.6 g Na_2_MoO_4_·2H_2_O, 4.0 g H_3_BO_3_, 4.0 g CoCl_2_·6H_2_O, 22.0 g ZnSO_4_·7H_2_O, 55.0 g CaCl_2_·2H_2_O and 37.5 g FeCl_3_·6H_2_O. Then, the chemostat culture was initiated after the depletion of glucose at the end of batch culture and continued at different dilution rates under the same medium condition. The cultures were carried-out at 30°C and pH 6.0 controlled by 24% NH_4_OH. Agitation and aeration were fixed at 800 rpm and 1 vvm, respectively, and the dissolved oxygen in the culture medium level was maintained above 10% air saturation during the entire culture period. Cultures were assumed to be in steady-state after at least 5 volumes changes.

### Analytical methods

The concentration of biomass was determined as dry cell weight (DCW). Culture samples were collected after achieving steady-state for different dilution rates. The collected samples were centrifuged and washed twice with ultrapure water and then the pellet was dried at 100°C. The culture supernatants were used for measuring the concentration of glucose, organic acids, glycerol and ethanol by high-pressure liquid chromatography (Gilson, Middleton, WI, USA) equipped with an HPX 87H column (Bio-Rad, Hercules, CA, USA), an ERC-7515A RI detector (ERC, Tokyo, Japan) and a UV detector (Youngin, Seoul, Korea). Analysis of carbon, nitrogen and hydrogen content was performed with a CHN analyzer (Carlo Erba Instruments, Rodano, Italy) of lyophilized cells as suggested by [79]. The ash content was determined by placing weighed quantities of lyophilized cells in ceramic crucibles, followed by incubation at 550°C for 6 hours. The exhaust gas leaving the fermentor was measured by LKM200A exhaust O_2 _and CO_2 _analyzer (Lokas, Daejeon, Korea). The carbon dioxide evolution rate (CER) and oxygen uptake rate (OUR) were deduced from the inlet and outlet carbon dioxide and oxygen compositions.

## Competing interests

The authors declare that they have no competing interests.

## Authors' contributions

BKSC performed the model reconstruction and drafted the manuscript. BKSC and SS performed the model analysis and simulations. JR, HL(1), JA and HL(2) performed the chemostat experiments. DYL coordinated and directed the project, and prepared the final manuscript. All authors have read and approved the final manuscript.

## Supplementary Material

Additional file 1 **Excel file contains metabolic reaction, metabolite and gene lists**. Click here for file

Additional file 2 **Word document contains details on the calculation of biomass composition, carbon balance and ATP maintenance requirement**. Click here for file
