# Supplementary material for: Genome-scale metabolic reconstruction and in silico analysis of methylotrophic yeast Pichia pastoris for strain improvement
Source: Microb Cell Fact. 2010 Jul 1;9:50. doi: 10.1186/1475-2859-9-50 (PMC2908565; doi:10.1186/1475-2859-9-50)
Supplement: Additional file 2 — Word document contains details on the calculation of biomass composition, carbon balance and ATP maintenance requirement. [file 1475-2859-9-50-S2.DOC]

Table of Contents

[***Pichia pastoris* biomass composition calculation 1**](#__RefHeading___Toc261605252)

[Overall cellular composition (Carnicer, et al., 2009) 1](#__RefHeading___Toc261605253)

[Amino acid composition (Carnicer, et al., 2009) 2](#__RefHeading___Toc261605254)

[Carbohydrates composition (Carnicer, et al., 2009) 2](#__RefHeading___Toc261605255)

[DNA composition 3](#__RefHeading___Toc261605256)

[RNA composition (Förster, et al., 2003) 3](#__RefHeading___Toc261605257)

[Lipid composition 3](#__RefHeading___Toc261605258)

[Growth associated ATP requirement for polymerization 5](#__RefHeading___Toc261605259)

[Additional biomass components 5](#__RefHeading___Toc261605260)

[Biomass composition summary 6](#__RefHeading___Toc261605261)

[Carbon content of cell mass 7](#__RefHeading___Toc261605262)

[Non-growth associated ATP maintenance (NGAM) requirement 8](#__RefHeading___Toc261605263)

[References 9](#__RefHeading___Toc261605264)

# *Pichia pastoris* biomass composition calculation

In the following calculations, general information such as molecular weight (MW) and chemical formula of each compound are obtain from the online PubChem database.

## Overall cellular composition (Carnicer, et al., 2009)

| **Components** | **g/gDCW** |
| --- | --- |
| Protein | 0.370 |
| Carbohydrates | 0.369 |
| RNA | 0.066 |
| DNA | 0.001 |
| Lipids | 0.062 |
| Others | 0.132 |

## Amino acid composition (Carnicer, et al., 2009)

The composition of aspartate cannot be distinguished from that of asparagine and this is also the same for glutamine and glutamate. Thus, we assume equal distribution of the composition within the pairs of amino acids. The molecular weight of the amino acids given in the following table excludes the weight of the water molecule that was lost during the formation of peptide bonds.

| **Amino acids** | **mol/100mol** | **MW (-H2O)** | **g/mol Protein** | **mmol/g Protein** | **mmol/gDCW** |
| --- | --- | --- | --- | --- | --- |
| Ala | 10.68 | 71.078 | 7.59 | 0.943 | 0.349 |
| Arg | 6.74 | 156.186 | 10.53 | 0.595 | 0.220 |
| Asn | 4.39 | 114.103 | 5.01 | 0.388 | 0.143 |
| Asp | 4.39 | 115.087 | 5.05 | 0.388 | 0.143 |
| Cys | 0.15 | 103.143 | 0.15 | 0.013 | 0.005 |
| Gln | 9.28 | 128.129 | 11.88 | 0.819 | 0.303 |
| Glu | 9.28 | 129.114 | 11.98 | 0.819 | 0.303 |
| Gly | 7.12 | 57.051 | 4.06 | 0.629 | 0.233 |
| His | 1.79 | 137.139 | 2.45 | 0.158 | 0.058 |
| Ile | 4.12 | 113.158 | 4.66 | 0.364 | 0.135 |
| Leu | 6.99 | 113.158 | 7.91 | 0.617 | 0.228 |
| Lys | 6.33 | 128.172 | 8.11 | 0.559 | 0.207 |
| Met | 0.77 | 131.196 | 1.01 | 0.068 | 0.025 |
| Orn | 1.53 | 115.154 | 1.76 | 0.135 | 0.050 |
| Phe | 3.03 | 147.174 | 4.46 | 0.268 | 0.099 |
| Pro | 3.67 | 97.115 | 3.56 | 0.324 | 0.120 |
| Ser | 6.43 | 87.077 | 5.60 | 0.568 | 0.210 |
| Thr | 5.77 | 101.104 | 5.83 | 0.510 | 0.189 |
| Trp | 1.40 | 186.210 | 2.61 | 0.124 | 0.046 |
| Tyr | 2.13 | 163.173 | 3.48 | 0.188 | 0.070 |
| Val | 5.58 | 99.131 | 5.53 | 0.493 | 0.182 |

## Carbohydrates composition (Carnicer, et al., 2009)

Carbohydrates, other than trehalose and glycogen were assumed to be glucan (13BDglcn) due to lack of detailed information.

| **Carbohydrates** | **g/100 gDCW** | **MW** | **mmol/gDCW** |
| --- | --- | --- | --- |
| 13BDglcn | 27.75 | 163.149 | 1.701 |
| Glycogen | 11.26 | 666.578 | 0.1689 |
| Trehalose | 0.33 | 342.296 | 0.0096 |

## DNA composition

GC content of Pichia pastoris is about 41.1% (De Schutter, et al., 2009).

| **DNA** | **MW** | **mol/mol DNA** | **g/mol DNA** | **mmol/gDCW** |
| --- | --- | --- | --- | --- |
| dAMP | 329.21 | 0.2945 | 96.951 | 0.00118 |
| dCMP | 305.18 | 0.2055 | 62.715 | 0.00082 |
| dGMP | 345.21 | 0.2055 | 70.940 | 0.00082 |
| dTMP | 320.19 | 0.2945 | 94.297 | 0.00118 |

## RNA composition (Förster, et al., 2003)

We assume the following RNA composition since total cellular RNA content of *P. pastoris* is similar to that of *S. cerevisiae*.

| **RNA** | **mmol/gDCW** |
| --- | --- |
| AMP | 0.051 |
| CMP | 0.050 |
| GMP | 0.051 |
| UMP | 0.067 |

## Lipid composition

The composition of lipids were calculated from data obtained from (Carnicer, et al., 2009; Wriessnegger, et al., 2009). Before we can evaluate the composition of lipids, we need to calculate the average molecular weight of a fatty acid chain based on data reported by Wriessnegger et al.:

| **Fatty acids (FA)** | **g/g FA** | **MW** | **mmol/g FA** | **mol % FA** |
| --- | --- | --- | --- | --- |
| Hexadecanoate (n-C16:0) | 0.155 | 255.42 | 0.607 | 0.167 |
| Hexadecenoate (n-C16:1) | 0.055 | 253.40 | 0.22 | 0.060 |
| Octadecanoate (n-C18:0) | 0.034 | 283.47 | 0.12 | 0.033 |
| Octadecenoate (n-C18:1) | 0.339 | 281.45 | 1.20 | 0.331 |
| Octadecadienoate (n-C18:2) | 0.288 | 279.44 | 1.03 | 0.283 |
| Octadecadienoate (n-C18:3) | 0.129 | 277.42 | 0.465 | 0.128 |

Taking the inverse of the sum of the values in the “**mmol/g FA**” column gives us the average molecular weight of a fatty acid chain to be 274 g/mol. Using the data by Carnicer et al., we can evaluate the molecular weights and composition of the phospholipids by adding the weight of the respective number of fatty acid chains to the phosphate-containing core structure of the phospholipids. Although Carnicer et al. reported the lumped composition of phphatidylinositol (PI) and phosphatidylserine (PS) as 0.4% w/w, we have decomposed the two based on the relative abundance of PI:PS (i.e. 10.8:4.4) reported by Wriessnegger et al. By taking into consideration that the total cellular lipid composition is 0.062 g/gDCW, we can calculate the individual lipid composition:

| **Lipid** | **g/g Lipid** | **Core MW** | **No. of FA** | **Lipid MW** | **mmol/g Lipid** | **mmol/gDCW** |
| --- | --- | --- | --- | --- | --- | --- |
| Triglycerol | 0.572 | 173.10 | 3 | 996 | 0.574 | 0.0356 |
| Cardiolipin | 0.003 | 508.22 | 4 | 1606 | 0.002 | 0.0001 |
| Phosphatidic acid | 0.001 | 226.08 | 2 | 775 | 0.001 | 0.00008 |
| Phosphatidylcholine | 0.100 | 312.23 | 2 | 861 | 0.116 | 0.00720 |
| Phosphatidylethanolamine | 0.052 | 269.15 | 2 | 818 | 0.064 | 0.0039 |
| Phosphatidylserine | 0.001 | 383.29 | 2 | 932 | 0.001 | 0.00008 |
| Phosphatidylinositol | 0.003 | 388.22 | 2 | 937 | 0.003 | 0.0002 |
| Sterols | 0.268 |  |  |  |  |  |

Sterol composition data given by Wriessnegger et al. is in µg/mg protein. These values are converted to biomass composition using the values of 0.268 g sterol/g lipid and 0.062 g lipid/gDCW given by Carnicer et al.

| **Sterols** | **µg/mg protein** | **g/g sterol** | **MW** | **mmol/g sterol** | **mmol/gDCW** |
| --- | --- | --- | --- | --- | --- |
| Episterol | 0.3 | 0.02 | 398.66 | 0.04 | 0.0007 |
| Ergosterol | 17.7 | 0.927 | 396.65 | 2.34 | 0.0388 |
| Fecosterol | 0.3 | 0.02 | 398.66 | 0.04 | 0.0007 |
| Lanosterol | 0.1 | 0.005 | 426.72 | 0.01 | 0.0002 |
| Zymosterol | 0.7 | 0.04 | 384.64 | 0.1 | 0.002 |

## Growth associated ATP requirement for polymerization

The ATP requirement for polymerization of each species is obtained from (Verduyn, 1991).

| **Polymer** | **g/gDCW** | **mmol ATP/g polymer** | **mmol ATP/gDCW** |
| --- | --- | --- | --- |
| Protein | 0.370 | 37.7 | 13.95 |
| Carbohydrate | 0.369 | 12.8 | 4.72 |
| RNA | 0.066 | 26.0 | 1.7 |
| DNA | 0.001 | 26.0 | 0.03 |

Total growth associated ATP requirement is 20.4 mmol ATP/gDCW.

## Additional biomass components

We include some essential metabolites in the biomass composition so as to qualitative account for the essentiality of their synthesis pathways. The composition of these metabolites is summarized in the following table:

| **Metabolite** | **mmol/gDCW** |
| --- | --- |
| Cyclic-AMP | 0.000001 |
| Chitin | 0.000001 |
| Coenzyme-A (CoA) | 0.000001 |
| FAD | 0.000001 |
| Glutathione | 0.000001 |
| NAD | 0.000001 |
| Protoheme | 0.000001 |
| Ubiquinone-6 | 0.000001 |
| Tetrahydrofolate | 0.000001 |
| Thiamin | 0.000001 |

It is noted that since the contribution of these metabolites is minute, we assume that they do not contribute quantitatively to any calculations with regards to cellular biomass.

## Biomass composition summary

| **Metabolite** | **mmol/gDCW** | **Metabolite** | **mmol/gDCW** |
| --- | --- | --- | --- |
| Ala | 0.349 | dGMP | 0.00082 |
| Arg | 0.220 | dTMP | 0.00118 |
| Asn | 0.143 | 13BDglcn | 1.701 |
| Asp | 0.143 | Glycogen | 0.1689 |
| Cys | 0.005 | Trehalose | 0.0096 |
| Gln | 0.303 | Episterol | 0.0007 |
| Glu | 0.303 | Ergosterol | 0.0388 |
| Gly | 0.233 | Fecosterol | 0.0007 |
| His | 0.058 | Lanosterol | 0.0002 |
| Ile | 0.135 | Zymosterol | 0.002 |
| Leu | 0.228 | Triglycerol | 0.0356 |
| Lys | 0.207 | Cardiolipin | 0.0001 |
| Met | 0.025 | Phosphatidic acid | 0.00008 |
| Orn | 0.050 | Phosphatidylcholine | 0.00720 |
| Phe | 0.099 | Phosphatidylethanolamine | 0.0039 |
| Pro | 0.120 | Phosphatidylserine | 0.00008 |
| Ser | 0.210 | Phosphatidylinositol | 0.0002 |
| Thr | 0.189 | Cyclic-AMP | 0.000001 |
| Trp | 0.046 | Chitin | 0.000001 |
| Tyr | 0.070 | Coenzyme-A (CoA) | 0.000001 |
| Val | 0.182 | FAD | 0.000001 |
| AMP | 0.051 | Glutathione | 0.000001 |
| CMP | 0.050 | NAD | 0.000001 |
| GMP | 0.051 | Protoheme | 0.000001 |
| UMP | 0.067 | Ubiquinone-6 | 0.000001 |
| dAMP | 0.00118 | Tetrahydrofolate | 0.000001 |
| dCMP | 0.00082 | Thiamin | 0.000001 |

# Carbon content of cell mass

The carbon content of 1 g of biomass can be evaluated based on the biomass composition and the chemical formulae of the respective biomass constituents. The carbon content of the lipids was calculated by adding the average carbon content of the fatty acids to the core structure. Consequently, the carbon content of one mole of each lipid molecules is as follows: cardiolipin, 83.2 C-mol/mol; phosphatidic acid, 40.1 C-mol/mol; phosphatidylcholine, 45.1 C-mol/mol; phosphatidylethanolamine, 42.1 C-mol/mol; phosphatidylserine, 48.1 C-mol/mol; phosphatidylinositol, 46.1 C-mol/mol; triacylglycerol 58.6 C-mol/mol. The carbon content of the other metabolites is given in the following table.

| **Metabolite** | **C-mol/mol** | **Metabolite** | **C-mol/mol** | **Metabolite** | **C-mol/mol** |
| --- | --- | --- | --- | --- | --- |
| Ala | 3 | Orn | 5 | dAMP | 10 |
| Arg | 6 | Phe | 9 | dCMP | 9 |
| Asn | 4 | Pro | 5 | dGMP | 10 |
| Asp | 4 | Ser | 3 | dTMP | 10 |
| Cys | 3 | Thr | 4 | 13BDglcn | 6 |
| Gln | 5 | Trp | 11 | Glycogen | 24 |
| Glu | 5 | Tyr | 9 | Trehalose | 12 |
| Gly | 2 | Val | 5 | Episterol | 28 |
| His | 6 | AMP | 10 | Ergosterol | 28 |
| Ile | 6 | CMP | 9 | Fecosterol | 28 |
| Leu | 6 | GMP | 10 | Lanosterol | 30 |
| Lys | 6 | UMP | 9 | Zymosterol | 27 |
| Met | 5 |  |  |  |  |

By multiplying the above numbers with the corresponding biomass composition values, we can calculate the carbon content of the *P. pastoris* biomass to be 36.35 C-mmol/gDCW. To determine validity of the constructed biomass synthesis reaction, we compare the computed biomass carbon content with experimental values and evaluate the *in silico* carbon balance. The *in silico* biomass carbon content evaluated to be 36.4 C-mmol/gDCW compares well with the value of 36.9 C-mmol/gDCW obtained from our chemostat experiment and the value of 35.9 C-mmol/gDCW reported by (Carnicer, et al., 2009).

Non-growth associated ATP maintenance (NGAM) requirement

The NGAM refers to the amount of ATP required by the cell even when it is not growing. This energy consumed for purposes other than the production of new cell material has been extensively reviewed (van Bodegom, 2007). In this study, we determined the NGAM requirement for our chemostat experiment using a conventional method of finding the y-intercept of the plot of glucose uptake rate against dilution rate (Pirt, 1982).


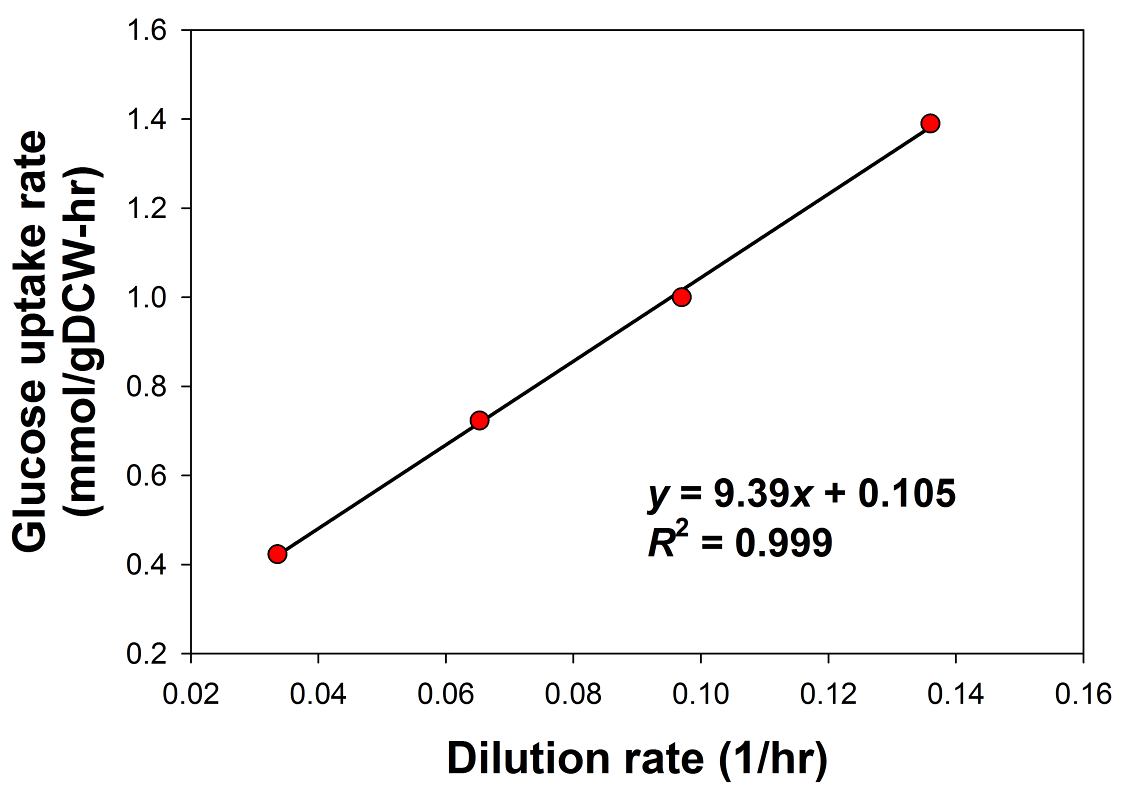


By maximizing ATP turnover under the glucose uptake constraint of 1 mmol/gDCW-hr, the ATP yield is evaluated as *Y*_ATP, max_ = 21.5 mol ATP/ mol glucose. Using this value and the y-intercept (0.105 mmol glucose/gDCW-hr), we can calculate the NGAM requirement to be about 2.26 mmol ATP/gDCW-hr.

References

Carnicer, M.*, et al.* (2009) Macromolecular and elemental composition analysis and extracellular metabolite balances of Pichia pastoris growing at different oxygen levels, *Microb Cell Fact.*, **8**, 65.

Carnicer, M.*, et al.* (2009) Macromolecular and elemental composition analysis and extracellular metabolite balances of Pichia pastoris growing at different oxygen levels, *Microb Cell Fact*, **8**, 65.

De Schutter, K.*, et al.* (2009) Genome sequence of the recombinant protein production host Pichia pastoris, *Nat Biotechnol*, **27**, 561-566.

Förster, J.*, et al.* (2003) Genome-scale reconstruction of the Saccharomyces cerevisiae metabolic network, *Genome Res*, **13**, 244-253.

Pirt, S.J. (1982) Maintenance energy: a general model for energy-limited and energy-sufficient growth, *Arch Microbiol*, **133**, 300-302.

van Bodegom, P. (2007) Microbial maintenance: a critical review on its quantification, *Microb Ecol*, **53**, 513-523.

Verduyn, C. (1991) Physiology of yeasts in relation to biomass yields, *Antonie Van Leeuwenhoek*, **60**, 325-353.

Wriessnegger, T.*, et al.* (2009) Lipid analysis of mitochondrial membranes from the yeast Pichia pastoris, *Biochim Biophys Acta*, **1791**, 166-172.
